# A Rat Model of Hemidystonia Induced by 3-Nitropropionic Acid 

**DOI:** 10.1371/journal.pone.0079199

**Published:** 2013-10-23

**Authors:** Huan-Guang Liu, Yu Ma, Da-Wei Meng, An-Chao Yang, Jian-guo Zhang

**Affiliations:** 1 Department of Neurosurgery, Beijing Tiantan Hospital, Capital Medical University, Beijing, China; 2 Beijing Neurosurgical Institute, Capital Medical University, Beijing, China; University of Iowa Carver College of Medicine, United States of America

## Abstract

**Objective:**

Secondary dystonia commonly presents as hemidystonia and is often refractory to current treatments. We aimed to establish an inducible rat model of hemidystonia utilizing 3-nitropropionic acid (3-NP) and to determine the pathophysiology of this model.

**Methods:**

Two different doses of 3-NP were stereotactically administered into the ipsilateral caudate putamen (CPu) of Wistar rats. Behavioral changes and alterations in the neurotransmitter levels in the basal ganglia were analyzed. We also performed an electromyogram, 7.0-T magnetic resonance imaging and transmission electron microscopy examination to determine the pathophysiology of the model.

**Results:**

In the CPu region, 3-NP produced mitochondrial cristae rupture, axonal degeneration, increased excitatory synaptic vesicles and necrosis. The extracellular concentrations of excitatory amino acids increased, whereas the inhibitory amino acids decreased in the CPu. Furthermore, an imbalance of neurotransmitters was found in other regions of the basal ganglia with the exception of the external globus pallidus. This study demonstrated that 3-NP administration results in CPu damage, and combined with a neurotransmitter imbalance in the basal ganglia, it produces specific neurobehavioral changes in rats. Right limb (contralateral side of CPu lesion) and trunk dystonic postures, shortened step length and ipsiversive dystonic posturing were observed in these rats. Furthermore, EMG recordings confirmed that co-contraction of the agonist and antagonist muscles could be seen for several seconds in right limbs.

**Conclusions:**

Stereotactic injection of 3-NP into the ipsilateral CPu of rats established an inducible model for hemidystonia. This effect might result from an imbalance of neurotransmitter levels, which induce dysfunctional activity of the basal ganglia mainly via the cortico-striato-GPi direct pathway. Symptoms in this model were present for 1 week. Activation of the cortico-striato-GPe indirect pathway and rebalance of neurotransmitters may lead to recovery. This rat model may be a suitable tool used to understand and further investigate the pathophysiology of dystonia.

## Introduction

Dystonia is the third most common movement disorder following Parkinson’s disease and essential tremor. Dystonia is typically defined as a syndrome of sustained or repetitive involuntary muscle contractions and abnormal postures and is classified as primary or secondary according to its etiology. Genetic factors contribute significantly to primary dystonia, where mutations in more than 17 genes have been implicated in its hereditary forms [1]. Secondary dystonia can be caused by metabolic, structural, infectious, toxic and inflammatory insults to the nervous system.

The pathophysiology of dystonia is poorly understood. However, patients with secondary dystonia commonly exhibit lesions within basal ganglia structures, including the caudate, putamen, globus pallidus, and thalamus [2]. Thus, dystonia has traditionally been considered a basal ganglia disorder. Clinically, the basal ganglia receive the most attention for its role in movement disorders. Abnormal neuronal activity in the basal ganglia has been reported during stereotactic surgery for deep brain stimulation (DBS) in dystonia patients [3-6].

Secondary dystonia commonly presents as hemidystonia; it is a unilateral clinical presentation of dystonia and is usually refractory to current methods of medical treatment. There have been few reports of documented clinical benefits with stereotactic interventions of the basal ganglia for hemidystonia [7-12]. In this context, it is thus necessary to determine the pathophysiology of this type of dystonia.

Given the inherent limits associated with conducting experiments in patients, animal models of dystonia are of considerable interest because they provide experimental paradigms for elucidating the mechanisms underlying this movement disorder [13]. However, the major challenge facing dystonia studies has been the limited availability of animal models of identified human dystonia, which faithfully mimic dystonic symptoms experienced by patients [14,15]. Although there have been several dystonic animals resulting from inherited mutations or animals in which dystonia was experimentally introduced that mimic the dystonic phenotype [14-16], secondary hemidystonia animal models are rare.

Basal ganglia have been described as one of the most commonly involved sites among patients of secondary dystonia. Within the basal ganglia, the striatum is the most prominent nucleus, serving as a major site of input and integration for cortical, thalamic, and midbrain afferents [17]. Interestingly, 3-nitropropionic acid (3-NP), a potent neurotoxin that interferes with mitochondrial respiration [18] induced segmental or generalized dystonia in patients [19,20]. Administration of 3-NP in rodents resulted in selective striatal lesions [18,21,22] and was associated with delayed onset dystonia, which is characterized by dystonia in the trunk and limbs [22,23]. Furthermore, 3-NP has been systemically administered in nonhuman species to establish an inducible model for dystonia for many years. 

The main aim of our study was to determine whether stereotactic administration of 3-NP into the striatum (caudate putamen, CPu) resulted in dystonic postures and thus more closely mimics the clinical phenotype of secondary hemidystonia observed in human patients. We analyzed changes in neurotransmitters in the basal ganglia, including the CPu, entopeduncular nucleus (GPi), globus pallidus (GPe) and subthalamas nucleus (STN) in a rat hemidystonic model. We also performed an electromyogram (EMG), 7.0-T magnetic resonance imaging (MRI) and transmission electron microscopy examination (TEM) to determine the pathophysiology of this hemidystonic animal model.

## Materials and Methods

All of the experiments were performed between 8:00 am and 3:00 pm, and in accordance with the Guidance for Animal Experimentation of the Capital Medical University and Beijing guidelines for the care and use of laboratory animals. The protocol was approved by the Committee on the Ethics of Animal Experiments of Capital Medical University (Permit Number: SYXK 2008-0005). All of the surgeries were performed under 10% chloral hydrate anesthesia, and all efforts were made to minimize animal suffering. The experimenters were blind to treatment effects.

### Elevated board cross test

As previously described [23], the animals were trained to cross an elevated board (120 cm long, 7 cm wide, 100 cm from the floor, painted black, with 5cm of each end inside a cage) to reach a platform in which their home cage was placed. The animals were trained for 1 week (3 trials/day), after which they were all able to cross the board without rearing or stopping. On the seventh day, three consecutive test runs were video-recorded using a camera placed laterally to the animals’ trajectory. The start line is 10cm away from the exit of the cage; the finish line is the entrance of the other cage. Steps were counted visually and the passing time was calculated with Ulead VideoStudio 10.0 (Taiwan, China). The locomotor behavior of each animal was subsequently evaluated on the basis of the passing time and the average length of steps. The motor performance was also assessed on the fifth day post-surgery. Based on previous report [23], we anticipate that 12 rats per group will be sufficient to generate reliable data.

### Surgery and behavior monitoring

Thirty-six adult, male Wistar rats weighing 200–300 g were used: (1) the high-dose group (n = 12), (2) the low-dose group (n = 12) underwent stereotactic administration of 3-NP, and (3) the control group (n = 12), which underwent stereotactic administration of saline. All of the rats of the high-dose group and low-dose group were anesthetized using 10% chloral hydrate (0.3 ml/100 g) and mounted in a stereotaxic frame (David Kopf Instruments, USA). Unilateral injections of 3-NP into the left CPu were made using the following coordinates: AP = 1 mm, L = 3 mm and V = 5.5 mm [24]. 3-NP (Sigma-Aldrich, St. Louis, MO, USA) at a dose of 4 μmol/1 μL (in phosphate buffer) for rats in the high-dose group and at a dose of 2 μmol/1μL for rats in the low-dose group, was injected over 5–10 min using a 5-μL Hamilton microsyringe. Rats in the control group were stereotaxically injected with saline. All of the rats awoke from the anesthesia within 1 h and were continuously monitored for their behavior. Dystonic posturing was recorded using a video camera placed 1.2 m above the observation cage.

### Electromyogram

Twenty-four hours after the 3-NP treated, 8 rats (high-dose) were used for the electromyogram (EMG) recordings. In 4 anesthetized rats, electrodes were surgically inserted into the triceps brachii and biceps brachii muscles in both forelimbs of each rat. In another 4 awake rats, 2 electrodes were surgically inserted into the triceps brachii and biceps brachii muscles in right forelimb, 2 electrodes were inserted into tibialis anterior and gastrocnemius muscles in right hindlimb of each rat. EMG signals were measured using a Neurosoft electrophysiology monitoring system (neuron spectrum 5, Russia).

### 7.0T MRI examination

Eight (high-dose, n = 4; low-dose, n = 4) 3-NP treated rats were scanned at 1, 3 and 7 days after 3-NP injection. MRI measurements were performed on a 7T Bruker ClinScan magnet with a 30 cm inner bore, capable of 290 mT/m in 250 μs (Bruker BioSpin MRI, Ettlingen, Germany). A quadrature birdcage transmit/receive radio-frequency (RF) coil (Morris Instruments, Canada) was employed. First, a sagittal scout image was taken to control for proper image alignment. The acquired coronal sections used for the analyses were taken perpendicular to a line connecting the superior end of the olfactory bulb and the superior end of the cerebellum with a field-of-view (FOV) of 3.0 × 3.0 cm^2^, matrix size 240 × 320 pixels. A total of 20 consecutive slices of 0.8 mm thickness each were acquired with TR = 3140 ms, TE = 41 ms, matrix size 240 × 320 pixels. 

### Assessment of neurotransmitters

Forty-eight adult, male Wistar rats weighing 200–300 g were used: (1) the 3-NP group (n = 24), which underwent stereotactic administration of 4 μmol/1 μl 3-NP, and (2) the control group (n = 24), which underwent stereotactic administration of saline. After the injection, a microdialysis-guided cannula with a stylet (Bioanalytical systems, USA) was implanted into the ganglia of each rat. The entopeduncular nucleus and globus pallidus in the rodents corresponded to the GPi and GPe in primates, respectively. The coordinates were [24]: CPu (AP = 1.0 mm, L = 3.0 mm and V = 4.5 mm), GPi (AP = -2.5 mm, L = 3.0 mm and V = 7.0 mm), GPe (AP = -1.2 mm, L = 3.2 mm and V = 5.5 mm), STN (AP = -3.6 mm, L = 2.5 mm and V = 7 mm). For the implantation, the cannulas were implanted into the CPu and GPi of 12 3-NP rats and 12 control rats, and in GPe and STN in the other rats. Three days after the surgery, the guided stylet was replaced with a microdialysis probe (CMA 12, Sweden). The probes were perfused with artificial cerebrospinal fluid (NaCl, 145 mM; KCl, 3.8 mM; MgCl_2_, 1.2 mM; CaCl_2_, 1.2 mM; pH 7.4) at a flow rate of 2 μl/min via a micro-syringe pump (CMA400, Sweden). The dialysis tubing was connected to a liquid swivel for collection from the awaked animal. The dialysis fractions were collected at 30 min intervals. A 2-h stabilization period was allowed before the collection of dialysis fractions for analysis. Next, 4 fractions were collected to analyze the neurotransmitters. Dialysis fractions were automatically collected with a refrigerated autosampler and stored at 80°C until further analysis. Seven days after 3-NP injection, the same procedure was performed in another 48 rats. According to our previous study which utilizing neurotransmitters analysis [25], 12 rats per group have sufficient powers to detect a difference.

The concentrations of amino acids in the dialysis samples were determined using high-performance liquid chromatography. Briefly, the samples or standards were derived from o-phtaldialdehyde; 20 μl of the resulting mixture was automatically loaded onto a Novapark C_18_ reverse-phase column (150 × 3.9 mM, 4 μm particle size; Waters) using a refrigerated autoinjector. The mobile phase consisted of NaH_2_PO_4_ (0.05 M, pH 6.8) with 20% methanol and the flow rate was 1 ml/min delivered using a Waters pump. The amino acid peaks were identified on the basis of the retention time. The extracellular concentrations of amino acids were estimated by rationing the peak areas of each amino acid and their respective external standards (analytical software: Empower).

### Histology

After completion of the experiment, all of the animals were perfused transcardially in deep anesthesia with 0.1 M phosphate-buffered saline and 4% paraformaldehyde. Next, the brains were removed, fixed in 4% paraformaldehyde and processed by Nissl staining for histological verification of the localization of the microdialysis probe on coronal sections (20 μm in thickness). Only rats with accurate placement were included in the data analysis.

### Transmission electron microscopic examination

The 3-NP-treated (high-dose, n = 4) rats were deeply anesthetized. These rats were transcardially perfused with PBS (0.1 M, pH 7.4) and were fixed with 500 ml of 2% glutaraldehyde and 1% formaldehyde in PBS. Tissue blocks of the CPu were extensively rinsed in 0.1 M PBS. Sections were osmicated (1% OsO4), dehydrated in a graded alcohol series to propylene oxide and flatly embedded in plastic in Epon 812. Ultrathin sections were collected on mesh nickel grids and examined using a Hitachi H-7650 electron microscope (Tokyo, Japan). The tissue preparations were imaged using a digital camera coupled to an electron microscope. 

### Statistical Analysis

Data of the elevated board test and neurotransmitters were represented as the mean ± SD. A one-way ANOVA was used to determine whether the neurotransmitters, passing time and step length differed between different groups followed by an LSD test for a comparison between two groups. Comparisons of the passing time and step length between pre- and post-operation were made using a paired t-test. Analyses were performed using the SPSS 17.0 software for Windows. An error probability of less than 0.05 was considered to be significant.

## Results

### Behavioral test

Motor deficits occurred in 3-NP treated rats, which had akinesia, right limbs and trunk dystonic postures (Video S1). Compared to the control group, 3-NP-treated rats demonstrated clear circling behavior turn to left (Video S2). No fixed deviated posturing was elicited in the control rats. However, the dystonic postures and circling behavior in 3-NP treated rats almost recovered on the 7^th^ day of post-operation (Video S3).

On the 5^th^ day of post-operation, the elevated board cross test revealed that rats treated with 3-NP displayed a significant decrease in step length and an increase in the passing time required to cross the platform compared to pre-operation (Table 1). The 3-NP-treated rats walked toward the lateral side of the board, which was consistent with the gait abnormalities and dystonic limbs. These symptoms became more severe following an increase in 3-NP dosage. These results also indicated that the time was significantly extended and the length of step was significantly shortened in rats that received a high-dose of 3-NP compared with rats in the low-dose group (Table 1). Because rats in the control group showed no dystonic posture, they can go through the board without retention．

**Table 1:** Analysis of variance between scores of both time and step length of passing the board in the board cross test on the 5th day post-operation and its paired t-test with the preoperative data (Mean ± SD).

	n	Time (s)					Step length (cm)			
		Pre-OP	Post-OP	*t*	*p*		Pre-OP	Post-OP	*t*	*p*
High-dose	12	1.67 ± 0.57	3.60 ± 1.62 ^a^, ^b^	3.893	0.001		11.41 ± 1.27	8.19 ± 0.73 ^a^, ^b^	7.615	0.000
Low-dose	12	1.53 ± 0.43	2.29 ± 0.31 ^a^	4.967	0.000		11.39 ± 1.47	9.24 ± 0.84 ^a^	4.399	0.000
Control	12	1.57 ± 0.59	1.61 ± 0.49	0.181	0.858		11.27 ± 1.36	11.18 ± 1.50	0.154	0.879
*F*		0.218	13.51				0.037	23.61		
*p*		0.805	0.000				0.964	0.000		

^a^ *p* < 0.05 vs. control group; ^b^ *p* <0.05 vs. low-dose group.

### EMG recording

**Figure 1:**
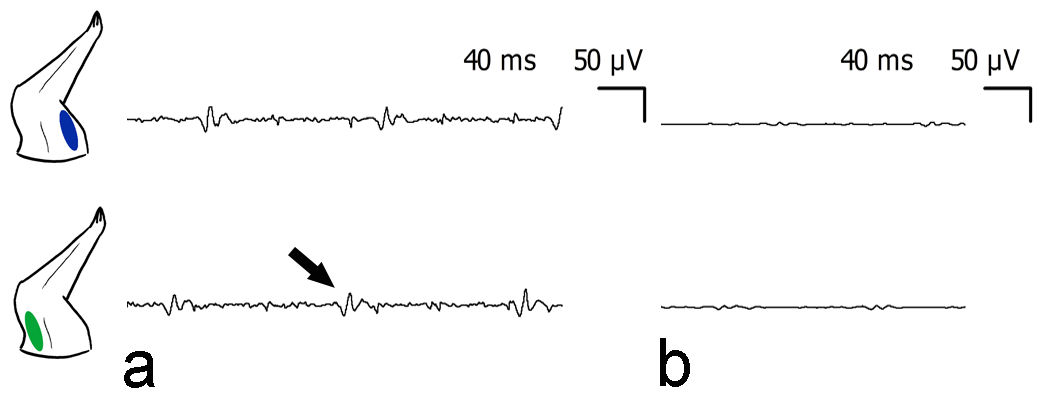
EMG recorded from agonist and antagonist triceps brachii (green) and biceps brachii (blue) muscles in the anesthetized rat, 24h after 3-NP injection. The signals confirmed that fibrillation potentials and positive sharp waves (black arrow indicated) simultaneously emerged in the agonist and antagonist muscles in the right forelimbs (a), while the EMG signals in the left forelimbs were normal (b).

**Figure 2:**
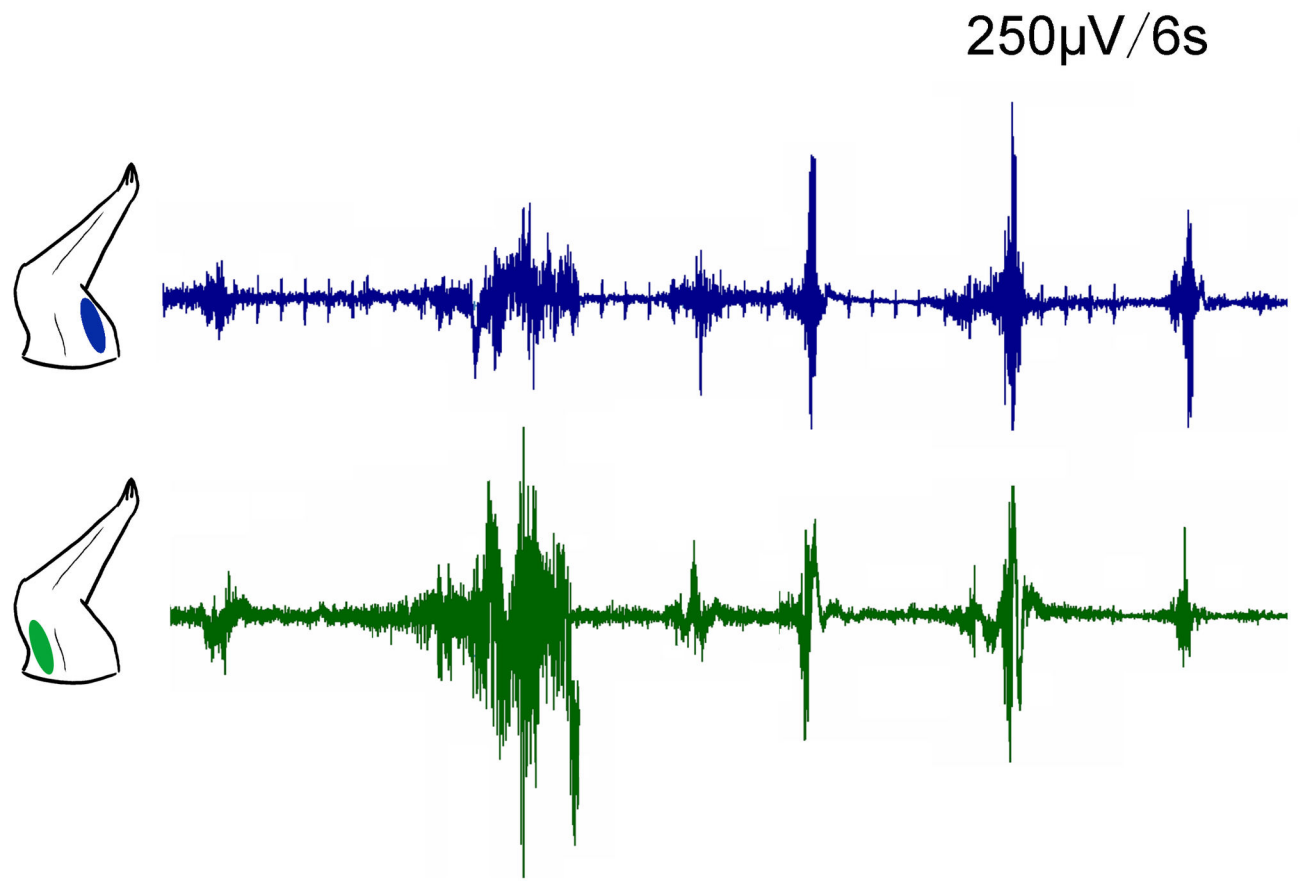
EMG recorded from agonist and antagonist triceps brachii (green) and biceps brachii (blue) muscles in the awake rat, 24h after 3-NP injection. Persistent co-contraction of the two muscles could be seen for several seconds.

**Figure 3:**
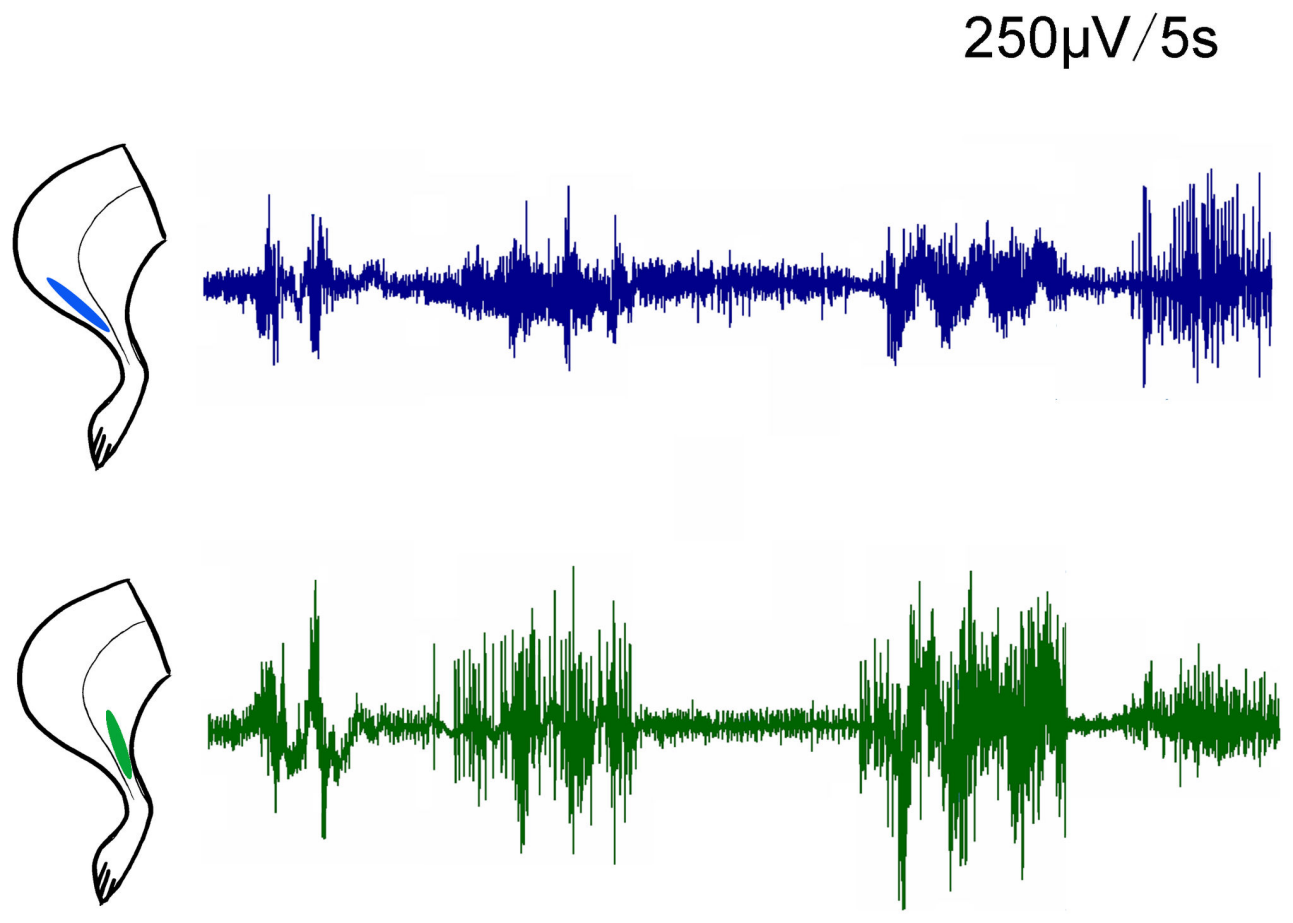
EMG recorded from agonist and antagonist triceps gastrocnemius (green) and tibialis anterior (blue) muscles in the awake rat, 24h after 3-NP injection. Persistent co-contraction of the two muscles could be seen for several seconds.

EMG recordings were performed in the triceps brachii muscle and biceps brachii muscle of the bilateral forelimbs. The EMG signals confirmed that the fibrillation potentials and positive sharp waves simultaneously emerge in the agonist and antagonist muscles of the right forelimbs; however, the EMG signals in left forelimbs were normal (Figure 1). EMGs also recorded from agonist and antagonist triceps brachii and biceps brachii muscles in right forelimb, and tibialis anterior and gastrocnemius muscles in right hindlimb of 3-NP rats. Co-contraction of agonist and antagonist muscles could be seen for several seconds in both right forelimb (Figure 2) and hindlimb (Figure 3).

### MRI scan

**Figure 4:**
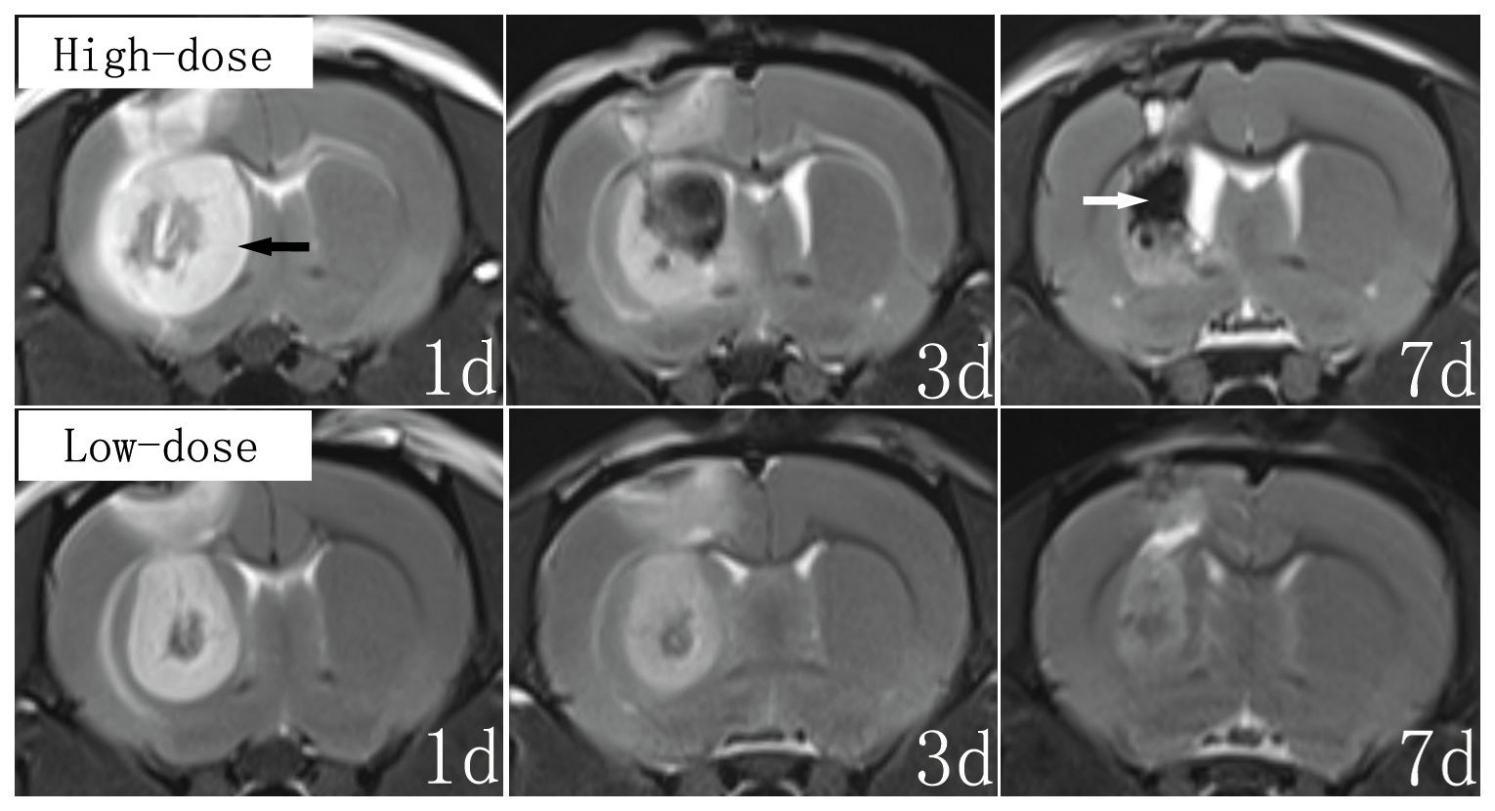
7.0-T MRI examinations after 3-NP injection. On the 1st day, the MRI results mainly showed edema (black arrow) that was associated with partial necrosis in the CPu region. On the 3rd day, the edema began to extenuate, while the necrosis was aggravated. On the 7th day, the MRI results mainly showed necrosis (white arrow). The lesions were more severe in the high-dose group compared to the low-dose group.

On the 1^st^ day postoperative 7.0-T MRI scans, the rats mainly showed edema, which was associated with partial necrosis in the CPu region. On the 3^rd^ postoperative day, the edema began to extenuate while the necrosis was aggravated. On the 7^th^ day, the rats mainly showed necrosis. The lesions were also more severe in the rats in the high-dose group compared to the rats in low-dose group (Figure 4).

### Imbalance between excitatory and inhibitory neurotransmitters

**Figure 5:**
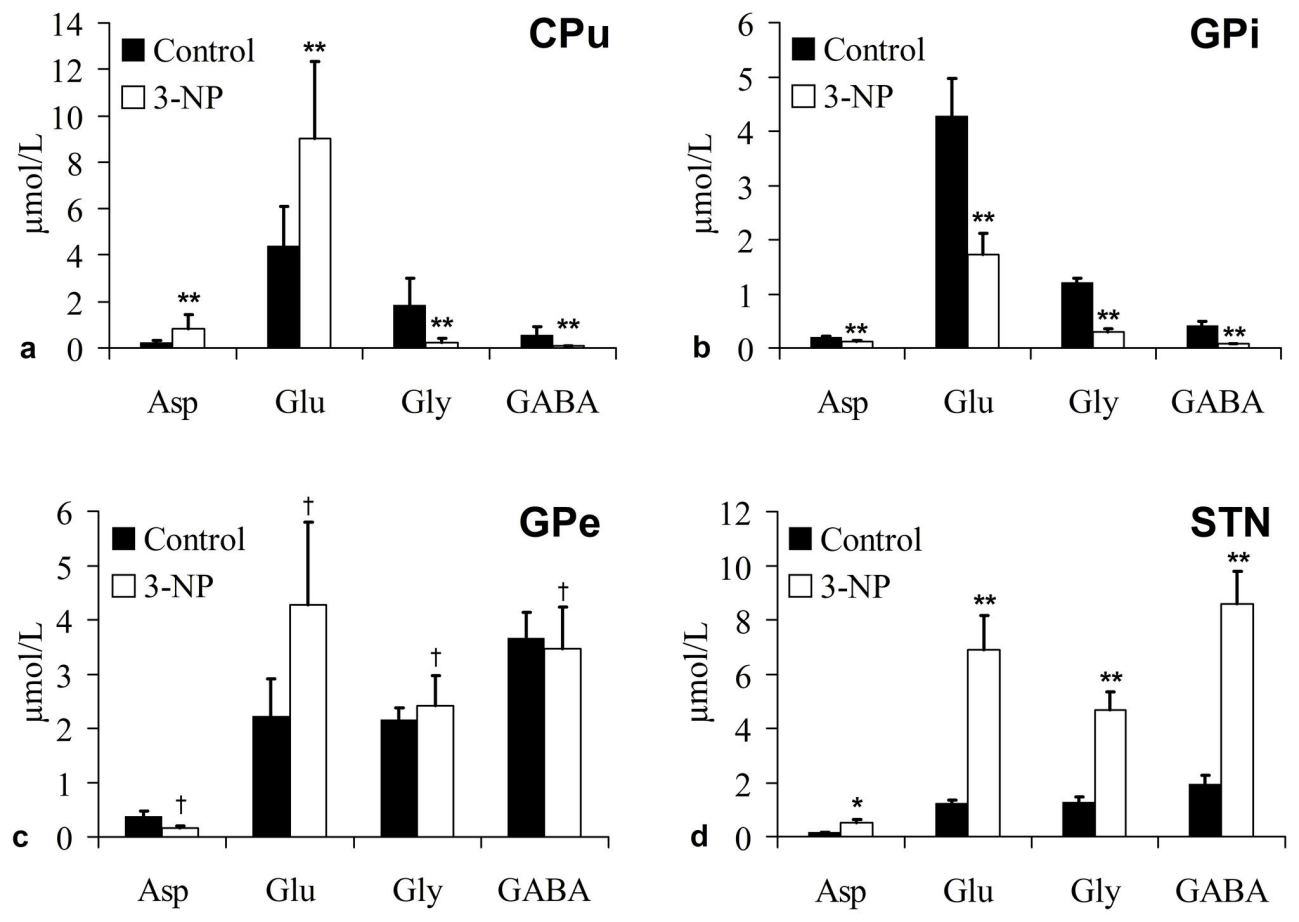
Concentrations of Asp, Glu, Gly and GABA in the CPu (a), GPi (b), GPe (c) and STN (d) in hemidystonia rats (4 μmol 3-NP injected, Day-3) compared to the control rats. The results are expressed as the mean ± SD; ^†^ *p* > 0.05, ^*^ *p* < 0.05, ^**^ *p* < 0.01 vs. control rats.

The levels of extracellular neurotransmitters were altered in the ganglia (Figure 5) on the 3^rd^ day of post-3-NP treatment. In the CPu, the levels of the excitatory extracellular neurotransmitters Asp (0.85 ± 0.57 µmol/L vs. 0.25 ± 0.06 µmol/L; p = 0.004) and Glu (9.03 ± 3.31 µmol/L vs. 4.38 ± 1.73 µmol/L; p = 0.000) were significantly increased in the 3-NP group compared to the control group, while the levels of inhibitory neurotransmitters Gly (0.24 ± 0.18 µmol/L vs. 1.84 ± 1.18 µmol/L; p = 0.001) and GABA (0.07 ± 0.03 µmol/L vs. 0.57 ± 0.34 µmol/L; p = 0.000) were significantly decreased. The extracellular neurotransmitters Asp (0.12 ± 0.06 µmol/L vs. 0.20 ± 0.05 µmol/L; p = 0.001), Glu (1.73 ± 1.34 µmol/L vs. 4.28 ± 2.41 µmol/L; p = 0.005), Gly (0.30 ± 0.18 µmol/L vs. 1.21 ± 0.30 µmol/L; p = 0.000) and GABA (0.07 ± 0.02 µmol/L vs. 0.41 ± 0.32 µmol/L; p = 0.004) in the GPi all significantly decreased; however, Asp (0.50 ± 0.47 µmol/L vs. 0.15 ± 0.04 µmol/L; p = 0.027), Glu (6.89 ± 4.35 µmol/L vs. 1.23 ± 0.45 µmol/L; p = 0.001), Gly (4.69 ± 2.21 µmol/L vs. 1.28 ± 0.59 µmol/L; p = 0.000) and GABA (8.58 ± 4.18 µmol/L vs. 1.96 ± 1.02 µmol/L; p = 0.000) all significantly increased in the STN. There was no significant difference in extracellular neurotransmitters in the GPe between the two groups (p > 0.05).

**Figure 6:**
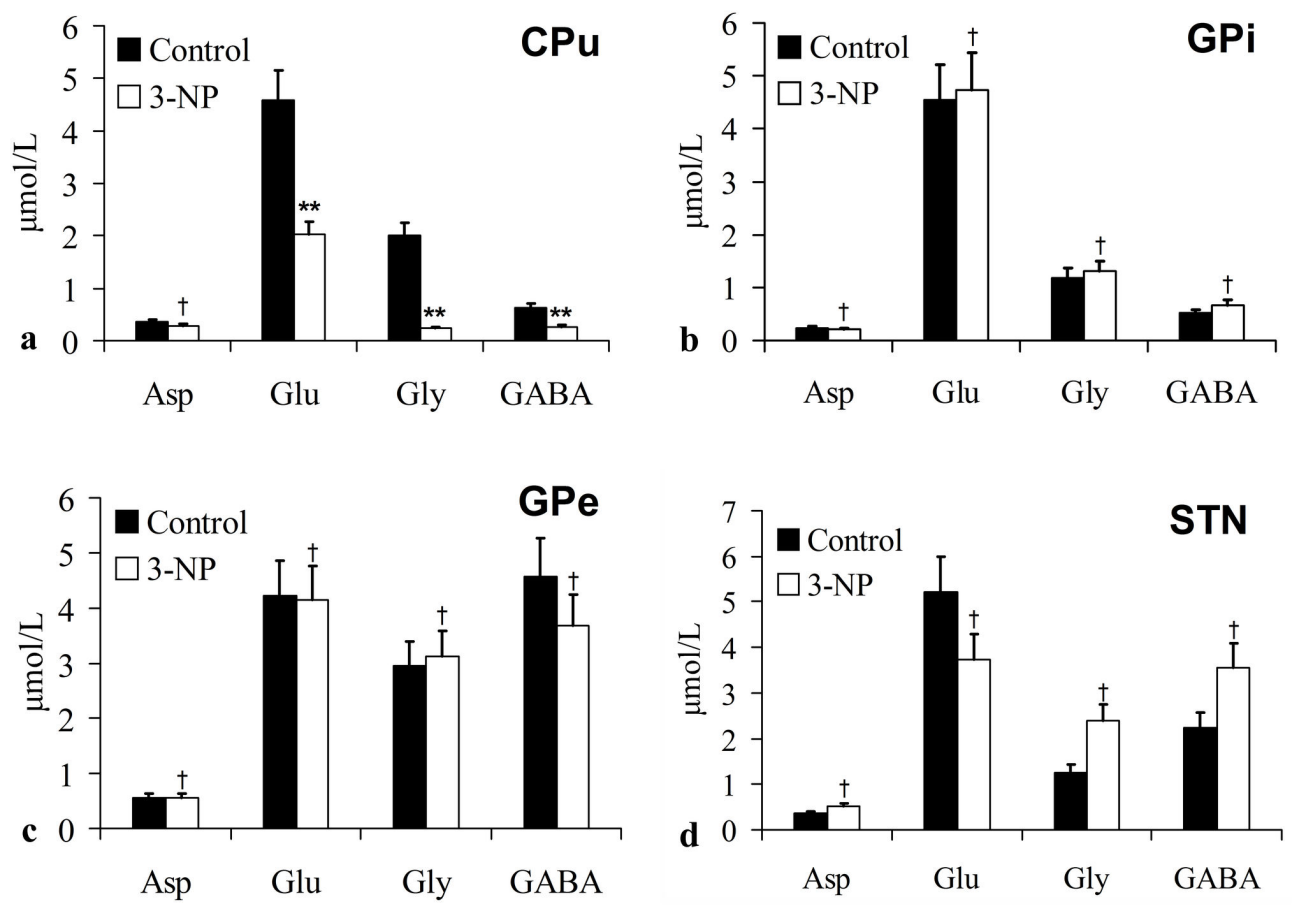
Concentrations of Asp, Glu, Gly and GABA in the CPu (a), GPi (b), GPe (c) and STN (d) in hemidystonia rats (4 μmol 3-NP injected, Day-7) compared to the control rats. The results are expressed as the mean ± SD; ^†^ *p* > 0.05, ^**^ *p* < 0.01 vs. control rats.

Seven days after 3-NP injection (Figure 6), in the CPu, the level of the excitatory extracellular neurotransmitter Glu (2.02 ± 0.25 µmol/L vs. 4.59 ± 0.58 µmol/L; p = 0.009) was significantly increased in the 3-NP group compared to the control group, similarly, the levels of inhibitory neurotransmitters Gly (0.23 ± 0.03 µmol/L vs. 2.01 ± 0.28 µmol/L; p = 0.006) and GABA (0.27 ± 0.03 µmol/L vs. 0.63 ± 0.06 µmol/L; p = 0.000) were significantly decreased. There was no significant difference in extracellular neurotransmitters in the GPi, GPe and STN between the two groups (p > 0.05).

### Transmission electron microscopic examination

**Figure 7:**
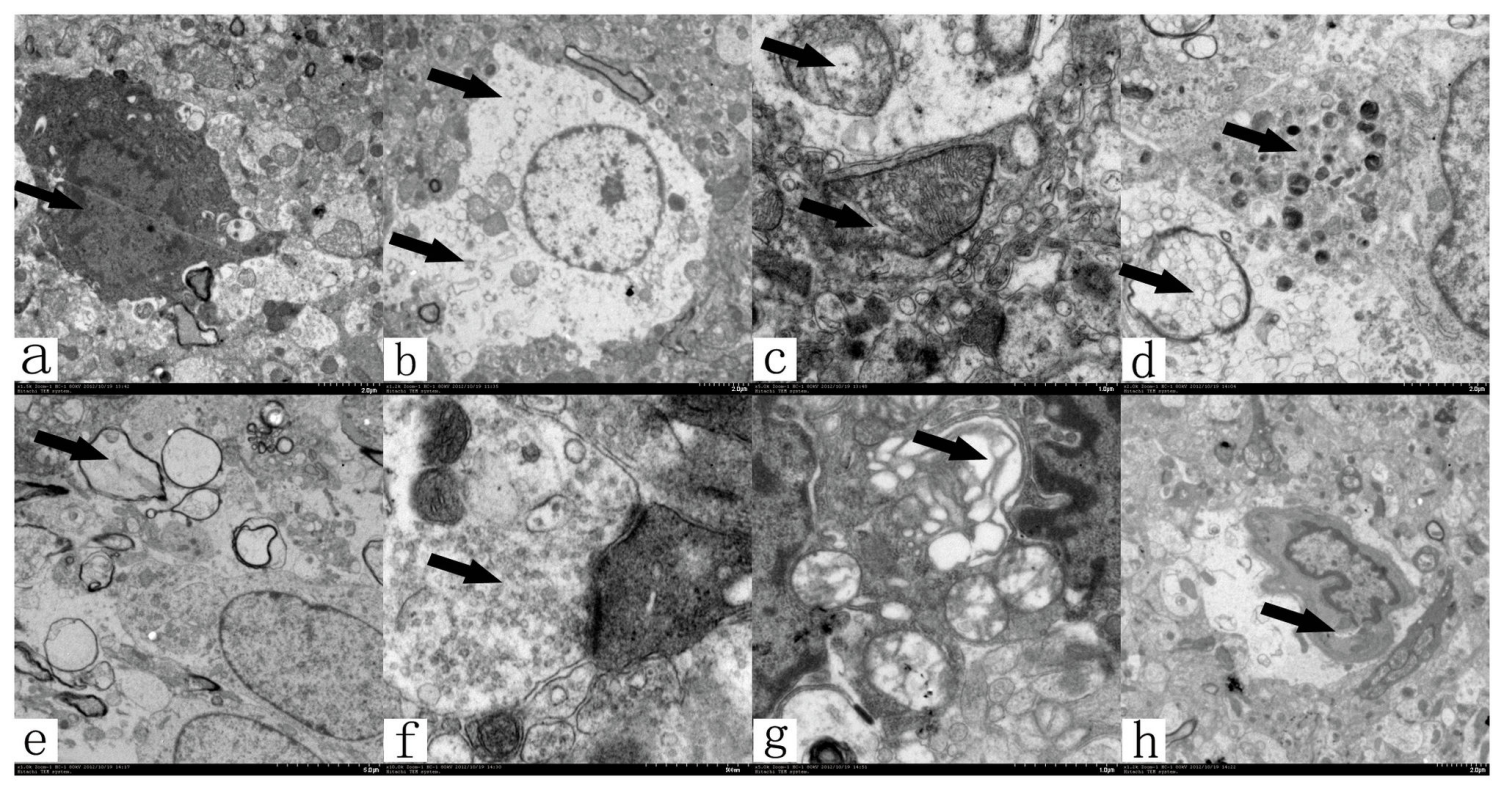
Transmission electron microscopy examinations after 3-NP injection. 3-NP treatment resulted in (a) pyknosis and (b) swelling in the neuron, as well as cell organelle disintegration. Findings further revealed the presence of (c) mitochondrial cristae rupture, (d) axonal degeneration and (e) cavitation, (f) increased excitatory synaptic vesicles, and (g) golgi vesicles expansion. Moreover, (h) glial foot process edema and the nucleus of endothelial cells increased resulting in vascular stenosis in the CPu region (black arrows indicate the lesions).

In the CPu sections, 3-NP treatment resulted in swelling and pyknosis in neurons combined with cell organelles disintegration. In addition, mitochondrial cristae rupture, axonal degeneration and cavitation, increased excitatory synaptic vesicles, and golgi vesicles expansion in the sections were observed. Moreover, the glial foot process edema and the nucleus of the endothelial cell increased, which resulted in vascular stenosis in the CPu region (Figure 7).

## Discussion

In this study, we present a rat model of hemidystonia induced by 3-NP, which was stereotactically injected into the CPu. We demonstrated that 3-NP injection resulted in CPu damage and neurotransmitter imbalance in the basal ganglia and produced specific neurobehavioral changes in rats. Akinesia, right limb (contralateral side of the CPu lesion) and trunk dystonic postures, as well as ipsiversive rotation, were observed in these rats. The EMG recordings confirmed that co-contraction of agonist and antagonist muscles occurred in the right limbs.

The primary mechanism of 3-NP activity involved irreversible inhibition of succinate dehydrogenase, a key mitochondrial enzyme of the electron transport chain [26]. Thus, 3-NP resulted in the depletion of ATP and energy failure. Peripheral administration of 3-NP in both rodents and primates causes selective lesions of the striatum [18,21,22,27-30] and dystonia. MRI results indicated that the dystonia was associated with lesions of the CPu [29-31]. In addition, focal destructive lesions of the posterior putamen also caused dystonia in primates [32]. In our MRI and TEM results, 3-NP treatment produced mitochondrial cristae rupture, axonal degeneration and cavitation, increased excitatory synaptic vesicles, Golgi vesicle expansion and necrosis in the CPu region. These pathophysiological findings were consistent with previous studies, which revealed energy failure and the initiation of excitotoxic events that subsequently resulted in neuronal cell death [26] and reactive astrogliosis [33]. Thus, 3-NP stereotactic injection into the ipsilateral CPu of rats may establish an inducible model for hemidystonia. For the 3-NP injection, we used two dosages. The MRI results revealed more severe CPu lesions in rats of the high-dose group compared to the rats of the low-dose group. In a behavioral test, the rats treated with the high-dose of 3-NP demonstrated more notable dystonic symptoms. Our results indicated that the time was significantly extended and the length of step was significantly shortened in rats that received a high-dose of 3-NP compared with rats in the low-dose group. These results were consistent with other previous studies. It has been reported that the severity of dystonia observed in nonhuman primates is consistent with the degree of striatal damage induced by 3-NP toxicity in humans and rodents [29]. In addition, the severity of dystonia correlates with the size of the striatal lesions [22,23].

The basal ganglia have been shown to play an important role in dystonia. Overt lesions in the basal ganglia as assessed using computed tomography or MRI were revealed in some dystonia patients [2,34,35]. Furthermore, functional MRI or positron emission tomography identified abnormal activity in the basal ganglia in many types of dystonia in the absence of overt lesions [36,37]. Thus, there may be correlations between basal ganglia dysfunction and dystonia. This hypothesis is also supported by neurosurgical treatments, which showed that dystonia was improved in patients following stereotactic surgery of the GPi [38-41]. However, how basal ganglia abnormalities produce dystonia is still unclear [42]. Genetic analyses, animal model studies and imaging studies in patients indicated disruptions in neurotransmitter communication in the striatum [15,43]. The striatum plays a pivotal role in the processing of neuronal activity via a circuit involving the cortex, striatum, and thalamus. In this circuitry, the striatum is the major site for motor-related inputs into the basal ganglia, and the GPi is the primary output nucleus [44,45]. The present study demonstrated that 3-NP produced an imbalance between excitatory (Glu, Asp) and inhibitory (GABA, Gly) neurotransmitters. The extracellular concentrations of Glu and Asp increased, whereas GABA and Gly decreased in the CPu regions of a rat model of hemidystonia compared to controls. Thus, overactivity of the excitatory pathways and reduced activity of the inhibitory pathways via the striatum may constitute one crucial mechanism involved in the initiation of hemidystonia. 

The projection neurons of the striatum, known as spiny projection neurons, integrate glutamatergic inputs from the cortex and thalamus and send GABAergic projections to neurons in downstream basal ganglia nuclei (GPe and GPi) [46]. These projections are divided into two pathways [47]; excitatory inputs to the striatum via the cortex inhibit GPi neurons using the direct pathway (cortico-striato-GPi-thalamus), whereas cortical activation of the striatum can excite GPi neurons using the indirect pathway (cortico-striato-GPe-STN-GPi-thalamus). We have shown that 3-NP injection reduced the extracellular concentration of inhibitory amino acids, particularly GABA in the striatum. Reduced GABA in the striatum could disinhibit the activity of GABAergic projection neurons and reduced neuronal activity in the GPi in the direct pathway. Thus, both decreases in inhibitory and excitatory amino acids in the GPi region were observed in this study. The reduced GPi activity excited thalamic and cortical neurons via a disinhibitory mechanism, and subsequently caused the dystonic symptoms. Decreased GABA levels were also detected in human dystonia. Markedly decreased levels of GABA were found in the sensorimotor cortex and striatum in patients with writer’s cramp [43] and in the basal ganglia in symptomatic dystonia [48]. Moreover, GABA-potentiating substances, such as benzodiazepines, are among the most effective therapeutics used to treat patients with dystonia [49], and intrastriatal injections of GABA-potentiating drugs significantly reduced the severity of dystonia [50]. On the other hand, enhanced Glu and Asp levels in the striatum may also contribute to the manifestation of dystonic behaviors in this study. The excitatory amino acids Glu and Asp play a critical role in regulating the physiological processes of movements [51]. It has been reported that cortico-striatal glutamatergic overactivity may contribute to the manifestation of dystonic attacks [52-54]. Our results are supported by previous studies [55,56], which revealed that 3-NP facilitated Glu release in the striatum, and the blockade of glutamate uptake potentiated 3-NP neurotoxicity. We also found increased excitatory synaptic vesicles in the TEM examination. Dystonic hamsters with paroxysmal generalized dystonia have also been shown to exhibit an increased presynaptic release probability at glutamatergic synapses [53]. Electrical or chemical stimulation of the frontal cortex resulted in enhanced concentrations of aspartate in the striatum of rats [57,58]. A previous study investigating the neurobiological mediation of spontaneous stereotypic behaviors indicated that rearing induced an elevation in striatal Glu and Asp concentrations [59]. Thus, the mechanisms responsible for the pathophysiology of the hemidystonia model include: (1) increased cortico-striatal glutamatergic activity, (2) disinhibited GABAergic projection neuronal activity, and (3) decreased inhibitory outputs of the GPi. The direct pathway plays a primary role in this hypothesis.

However, as our results showed, dystonic posturing and circling behaviors in the hemidystonia model were only present for 1 week. It is different from hemidytonia patients who have chronic refectory dystonic symptoms. Some study showed that striatal neurons project to both the GPe and GPi, the direct and indirect pathway might not be as segregated as previously thought [60]. In the experiments made by Kravitz et al., activation of direct and indirect pathways in the dorsomedial striatum regulates the pattern of motor activity. Direct pathway activation led to contraversive rotations, whereas indirect pathway activation yielded ipsiversive rotations. Their data also established a causal role for the indirect pathway in increasing freezing, decreasing locomotor initations, and inducing bradykinesia [61]. The symptoms of ipsiversive rotation and decreased locmotor also occurred in our dystonic rats. It suggests that a strong activation of the indirect pathway is occurring in this animal model. Thus, symptom relief may be due to increased excitatory input to the GPi via the indirect pathway, which is antagonistic to the effect of the direct pathway. This explanation appears supported by our neurotransmitter observations, which suggest that the excitatory and inhibitory amino acids in the STN were increased in this model on the 3^rd^ day after 3-NP injection, although we did not find any changes on the 7^th^ day. Additionally, in contrast with the 3^rd^ day after 3-NP treatment, striatal extracellular Glu level was significantly decreased on the 7^th^ day. Striatal neuronal necrosis, depletion of extracellular Glu and decreased cortico-striatal glutamatergic activity may be also an important reason for the recovery. Moreover, GABAergic projection neurons have recurrent collaterals back to the striatum [62]. An enhanced recurrent GABA release of projection neurons may counteract lower extracellular GABA levels within the striatum. Furthermore, Gly is not only an important inhibitory neurotransmitter in the central nervous system [63] but also a co-agonist with glutamate for the *N*-methyl-D-aspartate receptor. Striatal glycine can potentiate the function of the excitatory neurotransmitter Glu [64]. Thus, reduced extracellular Gly levels due to decreased excitatory overactivity in the striatum cannot be excluded in this study. 

There are some important limitations in this study. First, the vertical diameters of the GPi and STN in the rat are less than 1 mm [24]. Dialysis samples using a 2-mm probe from these nuclei may also consist of other parts of the basal ganglia. Thus, it cannot be excluded that other parts of the basal ganglia interfered with neurotransmitter changes in the GPi and STN. Innovatively designed probes with smaller and shorter membranes that will improve the spatial resolution and decrease tissue damage in microdialysis studies [65] can overcome this limitation. The second, although the necrosis occurred in the CPu region at 7^th^ day, dystonic postures and circling behavior in 3-NP treated rats lasted less than 7 days. Therefore, this rat model was different from hemidytonia patients. Both direct and indirect pathway were activated may be the most important reason. Moreover, fibrillation potential and positive waves in EMG are associated with denervation, it is possible that 3-NP entered the ventricle and influenced anterior horn of the spinal cord through the central canal.

## Conclusions

Our data suggested that 3-NP stereotactically injected into the ipsilateral CPu of rats could establish an inducible model of hemidystonia, and a high-dose of 3-NP could induce more reliable dystonic symptoms. 3-NP induced striatal lesions, which resulted in elevated Glu and Asp levels, which were consistent with the assumption that increased cortico-striatal activity of excitatory amino acids contributes to the manifestation of dystonic symptoms. In addition, decreased GABA levels enhanced the striatal inhibitory effects on the GPi via the direct pathway. Thus, one fundamental pathophysiological change of dystonia may include an imbalance of neurotransmitter levels, which may induce dysfunctional activity mainly via the cortico-striato-GPi direct pathway, resulting in an alteration of GPi inhibitory function on the thalamus. However, symptoms in this hemidystonia model were only present for 1 week. Ipsiversive rotation, decreased locmotor and increases in the amino acids levels in the STN region indicated that activity of the indirect pathway might also be increased. Activation of the cortico-striato-GPe indirect pathway and depletion of extracellular Glu may lead to recovery of this animal model. These findings may provide information toward a comprehension of the pathophysiology of dystonia.

## Supporting Information

Video S1 **Motor deficits occurred in 3-NP treated rat (4 μmol 3-NP injected, Day-3), which had akinesia, right limbs and trunk dystonic postures.**
(WMV)

Video S2 **Motor deficits occurred in 3-NP treated rat (4 μmol 3-NP injected, Day-3), which had ipsiversive rotation.**
(WMV)

Video S3 **Dystonic postures and circling behavior in 3-NP treated rat (4 μmol 3-NP injected) almost recovered on the 7^th^ day of post-operation.**
(WMV)
